# HbA1c changes in a deprived population who followed or not a diabetes self-management programme, organised in a multi-professional primary care practice: a historical cohort study on 207 patients between 2017 and 2019

**DOI:** 10.1186/s12902-024-01601-9

**Published:** 2024-05-20

**Authors:** Sarah Ajrouche, Lisa Louis, Maxime Esvan, Anthony Chapron, Ronan Garlantezec, Emmanuel Allory

**Affiliations:** 1 Department of General Practice, Univ Rennes, 2, Avenue du Pr Léon Bernard, RENNES Cedex, 35043 France; 2 CHU Rennes, Inserm CIC 1414 (Centre d’Investigation Clinique), Rennes, 35000 France; 3 CHU de Rennes, Univ Rennes, Inserm, EHESP (Ecole des Hautes Etudes en Santé Publique), Irset - UMR_S 1085, Rennes, 35000 France; 4 LEPS (Laboratoire Educations et Promotion de la Santé), University of Sorbonne Paris Nord, UR 3412, Villetaneuse, F-93430 France

**Keywords:** Diabetes self-management education, Deprived population, Diabetes mellitus, Primary healthcare

## Abstract

**Background:**

Diabetes self-management (DSM) helps people with diabetes to become actors in their disease. Deprived populations are particularly affected by diabetes and are less likely to have access to these programmes. DSM implementation in primary care, particularly in a multi-professional primary care practice (MPCP), is a valuable strategy to promote care access for these populations. In Rennes (Western France), a DSM programme was designed by a MPCP in a socio-economically deprived area. The study objective was to compare diabetes control in people who followed or not this DSM programme.

**Method:**

The historical cohort of patients who participated in the DSM programme at the MPCP between 2017 and 2019 (*n* = 69) was compared with patients who did not participate in the programme, matched on sex, age, diabetes type and place of the general practitioner’s practice (*n* = 138). The primary outcome was glycated haemoglobin (HbA1c) change between 12 months before and 12 months after the DSM programme. Secondary outcomes included modifications in diabetes treatment, body mass index, blood pressure, dyslipidaemia, presence of microalbuminuria, and diabetes retinopathy screening participation.

**Results:**

HbA1c was significantly improved in the exposed group after the programme (*p* < 0.01). The analysis did not find any significant between-group difference in socio-demographic data, medical history, comorbidities, and treatment adaptation.

**Conclusions:**

These results, consistent with the international literature, promote the development of DSM programmes in primary care settings in deprived areas. The results of this real-life study need to be confirmed on the long-term and in different contexts (rural area, healthcare organisation).

**Supplementary Information:**

The online version contains supplementary material available at 10.1186/s12902-024-01601-9.

## Introduction

Diabetes is a chronic disease that has doubled in prevalence in the last three decades [1] and is now one of the ten first causes of death worldwide [2]. Currently, 463 million people have diabetes worldwide (4.5 million in France) and this number could rise to 700 million by 2045 [3]. Diabetes incidence has increased dramatically, particularly that of type 2 diabetes mellitus that accounts for 90% of all cases [4]. Diabetes is associated with high morbidity index and altered quality of life [5–7]. Its prevalence has particularly increased in low-income and disadvantaged socio-economic groups [8–10], and even more in developed countries [11]. Its prevalence was twice as high in people receiving universal health coverage (UHC) [8] in whom it was also associated with worse glycaemic control [12] and more complications [13–15]. Higher diabetes prevalence was also found in some immigrant populations. For example, in metropolitan France, the risk of diabetes is 2.5 times higher in women who came from a North African country than in non-immigrant women [9]. Therefore, the population’s contextual and cultural characteristics need to be considered when developing preventive actions, such as Diabetes Self-Management (DSM) programmes [16, 17].

DSM education brings together the knowledge and skills that make people more aware about their health and their health choices by offering specific training, support and coaching [18]. DSM education enables people with diabetes to acquire and maintain skills to manage diabetes, resulting in quality of life improvement, increasing active role with the healthcare providers (HCP), and better adherence to treatment/follow-up and prevention of complications [19, 20]. The objective of DSM education is to make patients more autonomous and to produce a complementary effect to the usual pharmacological interventions [19]. It is an ongoing process, adapted to the disease course and the patient's lifestyle [21]. Although their effectiveness is acknowledged, particularly for type 2 diabetes mellitus [22–24], participation in DSM programmes in group settings is still limited among people with diabetes [25], especially in deprived populations. This difficult access is partly explained by their living conditions and socio-cultural background that complicate access to programmes and the will to change lifestyle habits [26]. Another explanation is that the current DSM programmes were not developed by taking into account the social and cultural background of the targeted populations [27].

The accessibility issues to DSM programmes and the obstacles to DSM practice are a major research topic [28, 29]. Furthermore, the fact that DSM education is mostly organised in hospitals [30, 31] may constitute an additional obstacle [32]. In 2014, in France, only 3.9% of self-management programmes were run in primary care settings, compared with 82% in a hospital structure [18]. Primary care now appears to be the preferred place for promoting access to care and reducing social inequalities in health [27]. Multi-professional Primary Care Practices (MPCP) bring together medical/paramedical professionals and social services around a common health project to improve inter-professional collaboration and access to care for the population [33]. Therefore, they seem suitable places for developing prevention programmes due to their accessibility based on their geographical position, relational proximity with the habitants, better cultural knowledge by the HCP and capacity to break down social isolation [34]. MPCPs are an opportunity to integrate DSM education in primary care and they could become reference structures in this field [35, 36].

In Rennes, the Villejean district is one of the five socio-economically deprived areas of the city. The median income is estimated at 670 euros (vs 1628 euros in the whole city), 38.3% of the population is unemployed, and 51% of < 20-year-old people receive UHC [37]. In 2015, 71 HCPs of this district decided to create the "Rennes Nord-Ouest" MPCP and developed a collective DSM programme for their patients with diabetes (supplementary files 1 and 2). In accordance with the recommendations, DSM programmes must be evaluated [18]. The value of this programme was initially demonstrated from the users’ point of view [34]. This qualitative study in 2020 also showed that in the first year of the DSM programme, participants were from nine different countries and 80% were considered as socio-economically deprived. This assessment must be continued by including quantitative biomedical parameters, as described in the international literature [38]. In Europe, several randomised controlled trials have demonstrated the benefit of group DSM for improving glycaemic control in non-deprived populations, such as the X-PERT study [39] and the DESMOND study [40]. In the United States, two randomised control trials carried out by community health workers in clinics found a significative effect of DSM programmes among socially deprived immigrant people with diabetes [41, 42]. However, we did not find any study on similar interventions for deprived people carried out in MPCPs.

The main objective of this study in a socio-economically deprived area was to compare diabetes control in a group that participated in a DSM programme run by an MPCP and in a group that did not receive this intervention.

## Methods

### Study design

This was an historical exposed/non-exposed cohort study to assess the effect of a DSM intervention in primary care, carried out by a MPCP located in a socio-economically deprived area of Rennes, France.

### Description of the intervention

The programme targeted ≥ 18-year-old people with diabetes to improve or develop self-care skills and change their eating habits. The DSM programme was designed and implemented by the "Rennes Nord-Ouest" MPCP, in the Villejean district, Rennes, France, in 2017. Patients were included in the programme upon suggestion by one of the MPCP HCPs involved in their care (e.g. general practitioner (GP), nurse, pharmacist, chiropodist), even if their own GP was not working at the MPCP. HCP of the MPCP recruited participants during their usual consultations. Refusal to participate was not recorded. Only interested patients had a BEPI (Bilan educatif partagé initial, patient-centred educational assessment) (supplementary file 3) with a HCP of the team before the DSM programme start to fix personal objectives that were used to prepare a personalized attendance programme to the different workshops.The programme consisted of seven to nine workshops that lasted 1–2 h and were held on weekdays between 9am and 5pm over a period of 1–2 months. The MPCP received annual funding from the local health authority (Agence régionale de santé) to cover the intervention running costs, and the training and remuneration of the involved HCPs.

### Exposed and non-exposed groups

The exposed group (receiving the intervention) included ≥ 18-year-old patients with type 1 or type 2 diabetes who were followed by at least one HCP in the MPCP and who participated in the DSM programme between 2017 and 2020. All the 75 patients who participated in the programme (at least BEPI completion) were eligible. If some had participated in more than one annual session, only their first participation was considered.

The non-exposed group included all the patients selected from the SOPHIA database of the GPs whose patients were in the exposed group. SOPHIA is a free diabetes support service set up by the French public health insurance in 2008 to offer remote coaching (emails, personal online space, and telephone follow-up with a nurse) adapted to the needs of people with diabetes in order to help them live better with their disease. This service was offered to all patients at the MPCP (i.e. people in the exposed and non-exposed groups). The SOPHIA database includes ≥ 18-year-old patients with type 1 and 2 diabetes who are registered with a GP, have long duration disease (LDD) status for diabetes, are affiliated to the public health insurance, and had at least three prescriptions for anti-diabetic drugs in the year of the intervention.

Each patient in the exposed group was randomly matched to two control patients based on sex (male or female), diabetes type (type 1 or type 2), year of birth (before 1960 or after; median calculated in the exposed group) and whether their GP was a MPCP member. The intervention date was the BEPI date.

The exclusion criteria for the exposed and non-exposed groups were: GP’s or patient’s refusal to participate in the study, patients unable to read and write in French, lack of follow-up during the study period (patient arrived at the practice after the intervention date, or left before), haemoglobinopathy that does not allow HbA1c monitoring, gestational diabetes, and drug-induced diabetes.

### Study endpoints

The primary outcome was glycated haemoglobin change (HbA1c in %) between 12 months before and 12 months after the intervention start date (i.e. the BEPI date).

Secondary outcomes were modifications in diabetes treatment, body mass index (BMI; in kg/m2), systolic and diastolic blood pressure (in mmHg), lipid profile (low density lipoprotein C, LDLc, in mmol/L), microalbuminuria, and screening for diabetic retinopathy between before and after the intervention.

### Data collection

Data were collected by two residents in general practice in 11 practices (21 GPs who followed the participants) after the intervention, between March and December 2021. Data were extracted from computerised medical records (consultations with clinical examination, laboratory work-up results, and specialist letters) from the practice professional software. Data were collected for the years 2017 to 2020, and as close as possible to the target dates (12 months before and 12 months after the intervention) to obtain at least two distinct values, particularly in terms of kidney function, lipid levels and microalbuminuria.

To characterise the two groups, each patient’s socio-demographic data (year of birth, sex, profession, education level, and socio-professional categories) and medical history (diabetes type and duration, other associated LDD) were collected. Concerning chronic treatment, prescriptions close to the target dates were identified to determine the diabetes treatments (metformin, other oral drugs, GLP-1 analogues, or insulin). Prescriptions for statins, angiotensin converting enzyme inhibitors, or related drugs were also retained.

Lastly, mentions of ophthalmological consultations (specialist’s letters or key words) were searched in the different consultations within the study interval.

### Statistical analysis

Patient characteristics were expressed as n (%) for categorical variables and mean ± standard deviation (SD) for continuous variables. For univariate comparison between (exposed and non-exposed) groups, the Student’s *t* or Mann–Whitney-Wilcoxon’s test was used for continuous variables and the χ2 or Fisher’s exact test for categorical variables.

Outcome changes over time were analysed using generalised linear mixed models. A sensitivity analysis was performed for the primary outcome using a model adjusted for sex, age, BMI, and education level. Multiple imputation was used to account for missing values. Fifty imputed datasets were created and combined using standard between/within-variance techniques. Statistical analyses were computed at the two-sided α level of 5% with SAS version 9.4 (SAS Institute, Cary, North Carolina, USA).

### Ethical aspects and legislation

This study was approved by the Rennes University Hospital ethics committee on 14 June 2021 (Number 21.77–2, supplementary file 5). It complied with the reference methodology MR-004 defined by the French committee on personal data protection (Commission Nationale Informatique et Libertés; CNIL) and with the European General Data Protection Regulation (GDPR).

## Results

**Fig. 1:**
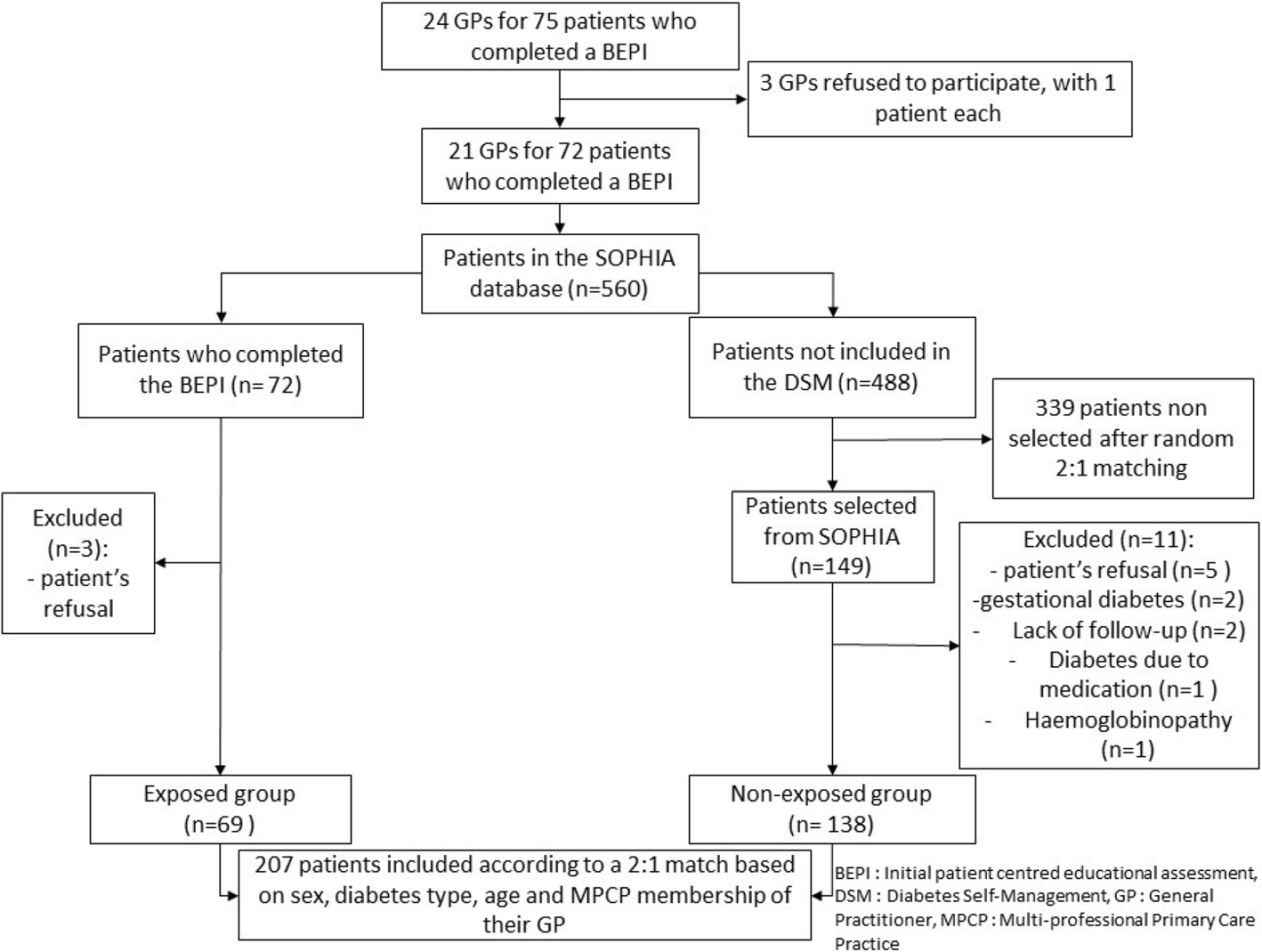
Flowchart

Among the 75 patients who completed a BEPI between 2017 and 2019, 24 GP’s were identified. Three GP’s refused to participate; each of them had one patient who had the BEPI. As three other patients with a BEPI refused to participate to the study, the exposed group was composed of 69 patients (Fig. 1). In the SOPHIA database, 488/560 patients followed by the GPs of the patients in the exposed group did not participate in the intervention. Therefore, a participation rate of 13% to the DSM programme could be estimated. Among them, 149 were selected by random 2:1 matching. After excluding 11 patients, 138 patients were included in the non-exposed group. With the 69 patients of the exposed group, 207 patients were included in the study.

### Description of the study population (Table 1)

**Table 1:** Comparison of baseline sociodemographic characteristics and comorbidities in the study population

	**Exposed group** **(** ***n*** ** = 69)**	**Non-exposed group (** ***n*** ** = 138)**	***p- value***
**Matching criteria and characteristics**			
Sex (missing *n* = 0)			*1.00*
-Female	44 (63.8%)	88 (63.8%)	
-Male	25 (36.2%)	50 (36.2%)	
Diabetes type (missing *n* = 0)			*1.00*
-Type 1	3 (4.3%)	6 (4.3%)	
-Type 2	66 (95.7%)	132 (95.7%)	
GP in the MPCP (missing *n* = 0)	62 (90.0%)	124 (90.0%)	*1.00*
Age in years (missing *n* = 0)	58 (± 12)	60 (± 14)	*0.40*
Age at diabetes diagnosis, in years (missing EG *n* = 1, NEG *n* = 11)	49 (± 12)	49 (± 13)	*0.89*
Diabetes known for < 1 year (missing EG *n* = 1, NEG *n* = 11)	13 (19.1%)	22 (17.3%)	*0.75*
**Education level** (missing EG *n* = 8, NEG *n* = 99)			
No schooling	2 (3.3%)	4 (10.3%)	*0.16*
Primary	19 (31.1%)	10 (25.6%)	
Secondary (middle school, high school)	32 (52.5%)	15 (38.5%)	
Higher education	8 (13.1%)	10 (25.6%)	
**Socio-professional categories** (missing EG *n* = 0, NEG *n* = 24)			
Farmers, craftsmen, shopkeepers	0 (0.0%)	0 (0.0%)	*0.77*
Executives, higher intellectual professions	0 (0.0%)	2 (1.8%)	
Intermediate professions	3 (4.3%)	7 (6.1%)	
Employees	12 (17.4%)	26 (22.8%)	
Workers	9 (13.0%)	10 (8.8%)	
Retired	29 (42.0%)	43 (37.7%)	
Not in employment^a^	16 (23.2%)	26 (22.8%)	
**LDD** (missing = 0)			
Presence of an additional LDD	29 (42.0%)	57 (41.3%)	*0.92*
Number of additional LDD	0.6 (± 0.9)	0.6 (± 0.8)	*0.89*
LDD for cardiovascular disorders	10 (14.5%)	24 (17.4%)	*0.59*
LDD for psychiatric disorders	10 (14.5%)	9 (6.5%)	*0.06*
LDD for neoplasia	5 (7.2%)	15 (10.9%)	*0.40*
LDD for respiratory disorders	4 (5.8%)	11 (8.0%)	*0.57*
LDD for neurological disorders	6 (8.7%)	9 (6.5%)	*0.57*
LDD for other disorders	7 (10.1%)	9 (6.5%)	*0.36*
**Comorbidities**			
Hypertension (missing EG *n* = 1, NEG *n* = 0)	46 (67.6%)	92 (66.7%)	*0.89*
Dyslipidaemia (missing *n* = 0)	35 (50.7%)	72 (52.2%)	*0.84*
Diabetic nephropathy (missing EG *n* = 2, NEG *n* = 0)	13 (19.4%)	25 (18.1%)	*0.82*
Diabetic retinopathy (missing EG *n* = 4, NEG *n* = 0)	7 (10.8%)	8 (5.8%)	*0.25*
BMI > 30 in kg/m^2^ (missing EG *n* = 2, NEG *n* = 19)	34 (50.7%)	56 (47.1%)	*0.63*

Continuous variables are expressed as mean (± standard deviation) and compared with the Student’s *t* or Mann–Whitney Wilcoxon test

Categorical variables are expressed as numbers (percentage) and compared with the Chi2 or Fisher’s exact test

A *p*-value < 0.05 was considered significant

*BMI* Body Mass Index, *EG* Exposed Group, *LDD* Long Duration Disease, *MPCP* Multi-professional Primary Care Practice, *NEG* Non-Exposed Group

^a^Unemployed people who have never worked, military, students, others

The analysis did not find any significant difference between groups concerning socio-demographic characteristics, age at diabetes diagnosis [49 (± 12) years for the exposed group and 49 (± 13) years for the non-exposed group], and percentage of patients with diabetes discovered < 1 year before the intervention date [*n* = 13 (19.1%) for the exposed group and *n* = 22 (17.3%) for the non-exposed group]. Education level and percentage of retired patients [*n* = 29 (42%) for the exposed group and *n* = 43 (37.7%) for the non-exposed group] were comparable between groups. Presence of another known LDD [*n* = 29 (42%) in the exposed group and *n* = 57 (41.3%) in the non-exposed group], mean number of LDDs per patient and their nature, and comorbidities (hypertension, dyslipidaemia, known diabetic nephropathy, known diabetic retinopathy or obesity) were not significantly different between groups.

### Pre-intervention data (Table 2)

**Table 2:** Pre-intervention data

	**Exposed group (** ***n*** ** = 69)**	**Non-exposed group (** ***n*** ** = 138)**	***p-value***
**Clinical data**			
Weight in kg (missing EG *n* = 2, NEG *n* = 6)	82.3 ± 14.8	83.6 ± 18.6	*p* = *0.61*
BMI in kg /m^2^ (missing EG *n* = 2, NEG *n* = 19)	31 ± 6.1	31 ± 6.0	*p* = *0.97*
SBP in mmHg (missing EG *n* = 1, NEG *n* = 3)	134.3 ± 13.1	132.4 ± 12.7	*p* = *0.32*
DBP in mmHg (missing EG *n* = 1, NEG *n* = 3)	76.8 ± 9.6	76.3 ± 9.6	*p* = *0.74*
**Chronic treatment**			
Metformin (missing *n* = 0)	45 (65.2%)	102 (73.9%)	*p* = *0.19*
Other oral treatment (missing EG *n* = 1, NEG *n* = 0)	30 (44.1%)	45 (32.6%)	*p* = *0.11*
GLP-1 analogue (missing EG *n* = 1, NEG *n* = 0)	12 (17.6%)	6 (4.3%)	***p*** ** = ** ***0.01***
Insulin (missing EG *n* = 1, NEG *n* = 0)	19 (27.9%)	23 (16.7%)	*p* = *0.06*
ACEI or ARA II (missing *n* = 0)	41 (59.4%)	68 (49.3%)	*p* = *0.17*
Statins (missing *n* = 0)	31 (44.9%)	58 (42.0%)	*p* = *0.69*
**Laboratory data**			
HbA1c in % (missing EG *n* = 2, NEG *n* = 2)	8.3 ± 2.2	7.1 ± 1.2	***p*** ** < ** ***0.01***
LDLc in mmol/L (missing EG *n* = 5, NEG *n* = 22)	2.8 ± 1.0	2.9 ± 0.9	*p* = *0.46*
GFR in mL/min/1,73m^2^ (missing EG *n* = 3, NEG *n* = 11)	86.4 ± 20.8	88.9 ± 21.5	*p* = *0.43*
Microalbuminuria test performed (missing *n* = 0)	56 (81.2%)	95 (68.8%)	*p* = *0.06*
Positive microalbuminuria (missing EG *n* = 13, NEG *n* = 43)	19 (33.9%)	17 (17.9%)	***p*** ** = ** ***0.02***
**Specialised follow-up**			
Ophthalmological consultation (missing EG *n* = 15, NEG *n* = 29)	39 (72.2%)	48 (44.4%)	***p*** ** < ** ***0.01***

Continuous variables are expressed as mean (± standard deviation) and compared with the Student’s *t* or Mann–Whitney Wilcoxon test. Categorical variables are expressed as numbers (percentage) and compared with the Chi2 or Fisher’s exact test

Positive microalbuminuria: defined as albuminuria ≥ 30mg/24h or albuminuria/creatinuria ratio ≥ 30mg/g

A *p* value < 0.05 was considered significant

*ACEI* angiotensin converting enzyme inhibitor, *ARAII* angiotensin II receptor antagonist, *BMI* body mass index, *DBP* diastolic blood pressure, *EG* exposed group, *GFR* glomerular filtration rate, *GLP-1* glucagon-like peptide 1, *HbA1c* glycated haemoglobin fraction A1c, *LDLc* low density lipoprotein c, *NEG* non-exposed group, *SBP* systolic blood pressure

Pre-intervention weight, BMI and blood pressure were not significantly different between groups. Among treatments, only prescription of GLP-1 analogues was higher in the exposed group than non-exposed group [*n* = 12 (17.6%) vs *n* = 6 (4.3%); *p* = 0.01]. Among laboratory data, the mean HbA1c level was significantly higher in the exposed than non-exposed group [8.3% ± 2.2 vs 7.1% ± 1.2; *p* < 0.01], and more patients had nephropathy with microalbuminuria in the exposed than non-exposed group [*n* = 19 (33.9%) vs *n* = 17 (17.9%); *p* = 0.02]. Adherence to the annual ophthalmological follow-up was higher in the exposed than non-exposed group [*n* = 39 (72.2%) vs *n* = 48 (44.4%); *p* < 0.01].

### Post-intervention changes (Table 3, Fig. 2)

**Table 3:** Variation of each variable in the exposed and non-exposed groups between before and after the intervention

	**Variable**	**Variation**		***p-value***
		**Exposed group**	**Non-exposed group**	
**Primary Outcome**	HbA1c *in %*	-0.73 [-1.13; -0.33]	0.35 [0.07; 0.63]	** < ** ***0.01***
	-HbA1c in patients with DT2	-0.75 [-1.17; -0.33]	0.38 [0.08; 0.67]	** < ** ***0.01***
	-HbA1c in patients with DT1	-0.46 [-2.04; 1.11]	-0.05 [-1.16; 1.06]	*0.63*
**Secondary Outcomes**	Metformin (*number of TT)*	1.12 [0.23; 2.00]	0.50 [-0.12; 1.14]	*0.27*
	Other oral treatment (*number of TT)*	-0.07 [-0.82; 0.67]	0.00 [-0.54; 0.56]	*0.86*
	GLP-1 analogue (*number of TT)*	0.27 [-0.58; 1.13]	0.99 [0.01; 1.98]	*0.28*
	Insulin (*number of TT)*	0.59 [-0.13; 1.31]	0.29 [-0.31; 0.90]	*0.54*
	ACEI/ ARA II (*number of TT)*	0.09 [-0.59; 0.78]	0.22 [-0.25; 0.70]	*0.77*
	Statins (*number of TT)*	0.02 [-0.65; 0.70]	0.19 [-0.29; 0.67]	*0.70*
	Weight (*kg)*	0.93 [-0.65; 2.52]	0.28 [-0.84; 1.42]	*0.51*
	SBP (*mmHg)*	3.77 [0.04; 7.50]	2.32 [-0.32; 4.96]	*0.53*
	DBP (*mmHg)*	3.11 [0.22; 5.99]	1.51 [-0.52; 3.56]	*0.37*
	BMI (*kg/m*^*2*^*)*	0.36 [-0.22; 0.95]	0.14 [-0.29; 0.59]	*0.57*
	LDLc (*mmol/L)*	-0.28 [-0.51; 0.05]	-0.16 [-0.33; 0.00]	*0.40*
	Microalbuminuria (*number of test performed)*	-0.22 [-1.03; 0.58]	0.31 [-0.40; 1.02]	*0.33*
	GFR (*L/min/1.73m*^*2*^*)*	-2.15 [-4.60; 0.29]	-4.48 [-6.21; 2.76]	*0.12*
	Ophthalmological consultation	-0.01 [-0.87; 0.85]	-0.25 [-0.79; 0.29]	*0.65*
**Sensitivity analysis**	HbA1c adjusted for sex, age, BMI, and education level	-0.72 [-1.13; 0.32]	0.36 [0.08; 0.64]	** < ** ***0.01***

A *p* value < 0.05 was considered significant

*ACEI* angiotensin converting enzyme inhibitor, *ARAII* angiotensin II receptor antagonist, *BMI* body mass index, *DBP* diastolic blood pressure, *DT1* type 1 diabetes, *DT2* type 2 diabetes, *GFR* glomerular filtration rate, *GLP-1* glucagon-like peptide, *HbA1c* glycated haemoglobin fraction A1c, *LDLc* low density lipoprotein c, *SBP* systolic blood pressure, *TT* Treatment

**Fig. 2:**
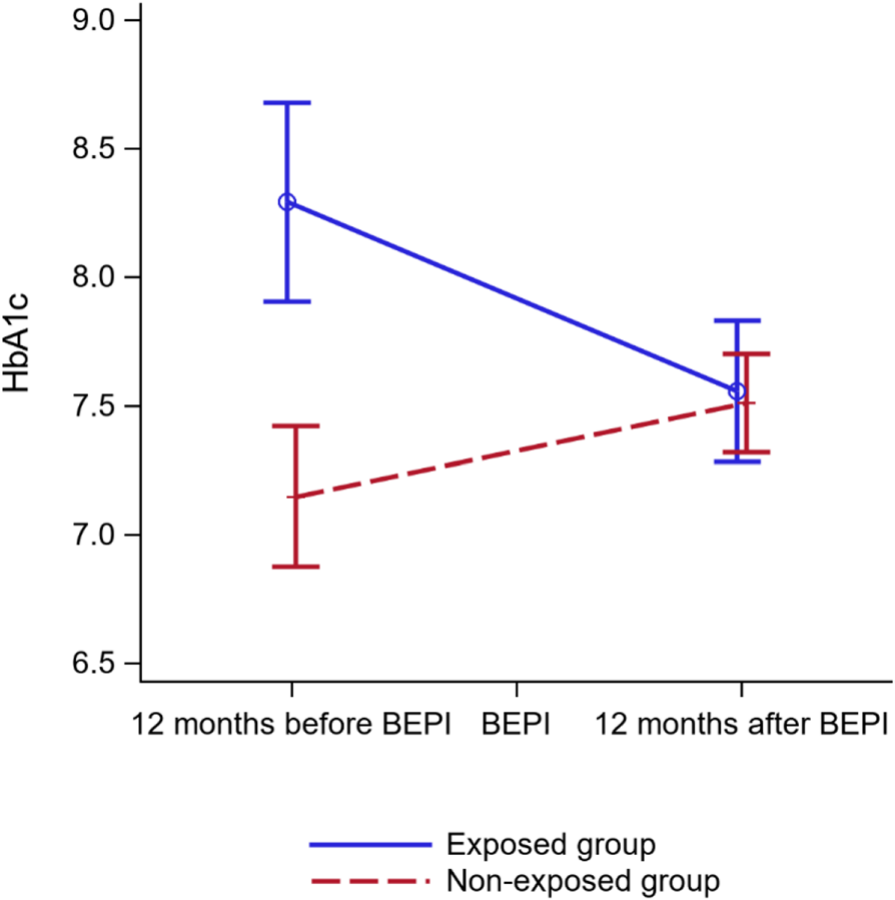
HbA1c (%) change over time (24 months) in the exposed and non-exposed groups

After the intervention, the mean HbA1c decreased by 0.73% [-1.13; -0.33] in the exposed group and increased by 0.35% [0.07; 0.63] in the non-exposed group (*p* < 0.01) (primary endpoint). All the secondary endpoints were similar between groups (supplementary file 6). In the secondary analyses, HbA1c change difference in the two groups after exclusion of patients with type 1 diabetes was still significant (*p* < 0.01) and remained also after the sensitivity analysis adjusted for sex, age, BMI and education level (*p* < 0.01).

## Discussion

The main result of our study is the significant difference in HbA1c change (*p* < 0.01) between the exposed group and the non-exposed group at 12 months post-intervention (i.e. DSM programme). This result is consistent with the literature. The systematic review by Odgers-Jewell et al. found that DSM education in groups efficiently reduced HbA1c by 0.3% at 12 months and up to 36 months [38]. Like in our study, there was no significant difference in BMI, blood pressure and LDLc change between exposed and non-exposed groups during the same period. The TIME randomised controlled trial on the long-term effectiveness of a programme for low-income populations in Houston community clinics found improvements in HbA1c at 12, 18 and even 24 months post-intervention [43]. Compared with the exposed group, HbA1c level in the non-exposed group (conventional medical follow-up) worsened. Similarly, the randomised controlled trial by Trento et al. [44] showed a progressive increase over 5 years in the HbA1c of controls compared with individuals receiving group DSM education in a hospital. In our study, the pre-intervention HbA1c and microalbuminuria were significantly higher in the intervention group, suggesting that patients who participated in the programme had more unbalanced and complicated diabetes. Hadjiconstantinou et al. found that patients with higher HbA1c (> 7%) benefit more from DSM programmes, as observed for our participants [29]. In this perspective article, the authors stressed that better outcomes were observed in groups that included participants with higher baseline HbA1c, younger age (< 65 years), and a higher proportion of ethnic minorities, like in our population. The lack of significant between-group difference in HbA1c and microalbuminuria after the intervention (supplementary file 6), combined with the analysis of variance for HbA1c, may indicate that the DSM intervention has a catch-up effect between groups, bringing both populations to same level. Indeed, while HbA1c decreased by 0.73% [-1.13; -0.33] in the exposed group, it increased by 0.35% [0.07; 0.63] in the non-exposed group (*p* < 0.01). Insulin prescription alone cannot explain this result because changes in insulin prescription were similar between groups (*p* = 0.54) and the HbA1c change difference remained also after the subgroup analysis adjusted for insulin prescription (*p* < 0.01). One hypothesis to be considered is that HCPs might have preferentially proposed the DSM programme to patients with badly controlled diabetes, although this was not an objective of the programme. In an interdisciplinary literature review, Carey et al. suggested the concept of "*proportionate universalism*" according to which health actions should be universal, but with a scale and intensity proportionate to the patients’ disadvantage level [45]. "*Proportionate universalism*" would be a way to move towards more equity in health by rebalancing situations without stigmatising population groups. Continuity of care in general practice allows practitioners to reduce social inequalities in health. Gray et al., in a systematic review of observational studies between 1996 and 2017, highlighted that increased continuity of care by doctors is associated with lower mortality rate in their patients [46]. Similarly, Sandvik et al. described the GP’s contribution to the life expectancy of their patients through the implementation of informal (access to all the patient's information), longitudinal (transcending the various disease episodes), and interpersonal (the relationship of trust established between patient and GP) continuity [47].

Another important finding in our study was the significant higher adherence to the ophthalmological follow-up in the exposed group than in the non-exposed group (72.2% versus 44% before the intervention and 72% versus 38.1% after the intervention). This may be explained by a closer follow-up of patients in the exposed group by their GP/other HCPs. However, this does not seem to have had an effect on baseline HbA1c that was higher in the exposed group. Additionally, our exposed group may have had a lower level of health literacy (i.e. the set of individual and environmental conditions for a patient to understand and process health information) [48]. This could explain why the GP better followed these patients and, for instance, might have been more likely to ask the secretary of the practice to organise an appointment with the specialist rather than delegating this task directly to the patient. According to the French national health council (Haut Conseil de la Santé Publique; HCSP), "*people with low literacy level are 1.5 to 3 times more likely to be in unfavourable health conditions than people with higher literacy level*" [27]. This could explain the initial difference in HbA1c level between groups. A qualitative study on the health literacy level of participants in a DSM programme in a socio-economically deprived area of Montpellier (south of France) highlighted the diversity of health literacy profiles that coexisted in that area [49]. Moreover, low health literacy is more likely to be observed among people with low income, belonging to ethnic minorities, or migrant populations [27]. Our exposed group included mainly patients from a practice in an area with elevated socio-economic difficulties and consequently people with more precarious profiles.

### Strengths and limitations

To our knowledge, this is the first French study that evaluated the effect on HbA1c of a DSM intervention carried out by an MPCP in a socio-economically deprived area. Another of its strengths is that patients were from different general practices in this deprived area and their medical records were fully accessible. Moreover, our exclusion criteria included absence of follow-up during the study period or the presence of a pathology that did not allow HbA1c monitoring. The aim was to optimise data collection, especially for the primary outcome (HbA1c changes). Our study also has several limitations including missing data, potential residual cofounding, and potential selection bias. First, data were missing for some variables, especially education level and participation rate. Education level is not routinely collected in medical records. We assumed that this variable was missing at random and consequently we used the multiple imputation method to deal with this issue. The obtained results were in accordance with the main analysis. Second, other information (e.g. private health insurance status, marital and family situation, country of birth, understanding of written French, financial situation) was not present in the medical records. These missing data would have allowed matching the two groups also for these socio-economic variables. In our opinion, to develop research in primary care in France, the healthcare organisation needs to think how the patients’ socio-economic data could be collected using the GP’s professional software tools. Moreover, the study retrospective nature did not allow collecting other potential cofounding variables, for instance participation in other DSM programmes or individual data about deprivation for both groups. In addition, we used a logistic regression to take into account potential confounding factors collected in our study. Alternatively, we could have used a propensity score to take into account the non-random allocation of the intervention in our study. However, the performance of these two methods is similar in observational studies [50–52]. Lastly, we did not know why some patients with diabetes followed at this MPCP did not participate in the DSM programme (refusal rate and reasons for this choice). Therefore, we could not exclude, in addition to a possible reversion to the mean, a selection bias because our exposed group may constitute a subgroup of the population with diabetes more committed to better control their HbA1c.

## Conclusion

Our findings suggest that HbA1c improved after participation in a DSM programme led by an MPCP in a socio-economically deprived area. This needs to be confirmed by a prospective study, but it should already encourage the development of DSM targeted to deprived populations in primary care.

### Supplementary Information

Supplementary Material 1.

## Data Availability

The datasets used and analysed in the current study are available from the corresponding author on reasonable request.

## Abbreviations

ACEI: Angiotensin Converting Enzyme Inhibitor
ARA II: Angiotensin II Receptor Antagonist
ARS: Agence Régionale de Santé (Local Health Authority)
BEPI: Bilan Educatif Partagé Initial (Initial patient-centred educational assessment)
BMI: Body Mass Index
CKD-EPI: Chronic Kidney Disease Epidemiology
CNIL: Commission Nationale de l’Informatique et des Libertés (French Committee on Data Protection)
CSS: Complémentaire Santé Solidaire
DBP: Diastolic Blood Pressure
DSM: Diabetes Self-Management
GDPR: General Data Protection Regulation
GFR: Glomerular Filtration Rate
GLP-1: Glucagon-Like Peptide-1
GP: General Practitioner
HAS: Haute Autorité de Santé (French Health Authority)
HbA1c: Glycated Haemoglobin fraction A1c
HCP: HealthCare Provider
HCSP: Haut Conseil de la Santé Publique (National Health Council)
INSEE: Institut National de la Statistique et des Etudes Economiques (National institute of statistic and economic studies)
LDD: Long Duration Disease
LDLc: Low Density Lipoprotein C
MPCP: Multi-professional Primary Care Practice
T2DM: Type two Diabetes Mellitus
RCT: Randomised Controlled Trial
RGPD: Règlement Général sur la Protection des Données (General data protection framework)
SBP: Systolic Blood Pressure
UHC: Universal Health Coverage
