# Supplementary material for: HbA1c changes in a deprived population who followed or not a diabetes self-management programme, organised in a multi-professional primary care practice: a historical cohort study on 207 patients between 2017 and 2019
Source: BMC Endocr Disord. 2024 May 20;24:72. doi: 10.1186/s12902-024-01601-9 (PMC11103828; doi:10.1186/s12902-024-01601-9)
Supplement: Supplementary file 1 — Supplementary Material 1. [file 12902_2024_1601_MOESM1_ESM.docx]

**Supplementary files**

[Supplementary file 1 - Objectives of the DSM programme 2](#_Toc129094223)

[Supplementary file 2 – Information on the DSM programme 3](#_Toc129094224)

[Supplementary file 3 – Initial patient-centred educational assessment (BEPI) 5](#_Toc129094225)

[Supplementary file 4 - Information letter for research participation 7](#_Toc129094226)

[Supplementary file 5 - Agreement of the University Hospital Ethics Committee 11](#_Toc129094227)

[Supplementary file 6 – Post-intervention Data 12](#_Toc129094228)

# Supplementary file 1 - Objectives of the DSM programme

**The objectives of the DSM programme for the patients were:**

- To acquire or maintain the skills/knowledge to better manage their life with diabetes, in order to become actors of their health.
- To become more independent through the capacity to define personal objectives concerning diabetes management, to identify what must be done to reach this objective, and the inner and external resources to be mobilized (at the level of primary and secondary care).
- To maintain, improve and preserve their quality of life through a better acceptance of their disease, and a better expression of their feelings about their disease, their family/friends or other people with diabetes.

# Supplementary file 2 – Information on the DSM programme

**The programme included**

1. **Initial patient-centred educational assessment (BEPI)**, established during a meeting between the patient and an MPCP healthcare professional trained in DSM. This meeting allows identifying the patient’s priorities and defining together one or more short or medium-term objectives. Then a personalized education programme is established based on the resources identified by the patient, and also on the resources that may be proposed by the MPCP or its partners.

This personalized programme is validated and signed by the patient and the healthcare professional present at the meeting. The programme is then sent to the different speakers/facilitators in the groups that will be attended by the patient and to the healthcare professionals implicated in the patient’s follow-up (particularly general practitioner).

1. **Group education activities proposed to the patient.** The MPCP proposes a cycle of seven 90-minute workshops that include about ten people, twice per year. The proposed themes are:
2. **Diet** (three sessions). The first session is focused on the patients’ perceptions concerning carbohydrates. The workshop is co-led by a nurse trained in DSM and a physician nutrition specialist from the MPCP. The second session focuses on assessing the knowledge on fruits and vegetables in the patients’ diet, by promoting a varied diet. The third session is a cooking workshop to develop the patient’s independence in preparing their meals, after having bought the ingredients at the local market. It finishes with a meal shared by patients and healthcare professionals. These workshops were developed by taking into account the cultural diversity and socioeconomic status of the local population.
3. **Knowledge of the disease** (two sessions). The objectives of the first session are to explore the patients’ perception of their disease to allow them to understand how their disease works, the different actions, and their place in the disease management. The second session is focused on the diabetes pharmacological treatments. These workshops are led by two general practitioners trained in DSM.
4. **Physical activity** (one session). This 90-minute workshop tries to explore the patients’ perceptions concerning physical activity and its potential benefits for diabetes. The speaker, a physiotherapist, defines with the patients simple physical exercises that they can do at home.
5. **Foot care.** The local podiatrist discusses about the importance of foot care with the patients, of performing simple daily foot hygiene measure and of monitoring the appearance of signs that may suggest something serious and that should lead the patients to see their doctor.
6. **The diabetes kit**. In this session, general practitioners explain how to use the material required for a patient with diabetes and work on the concept of easy of care. The idea is to adapt with the patient the choice of material.
7. **Final patient-centred educational assessment (BEPAFI)** is performed by the same person who did the initial assessment, in a face-to-face meeting, at the end of the therapeutic education cycle. It allows determining whether the objectives have been attained and defining, if needed, a new personalized programme. It is focused on highlighting the presence of a process of change, an improvement of the capacity to participate in the disease management, to mobiliser resources in the daily life. It also evaluates the improvement in the patient’s quality of life. A summary of this assessment is sent to the treating physician by secure e-mail.

# Supplementary file 3 – Initial patient-centred educational assessment (BEPI)

# Supplementary file 4 - Information letter for study participation


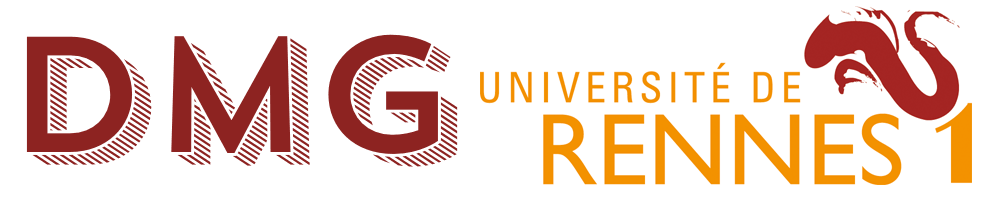
**INFORMATION LETTER**

**FOR STUDY PARTICIPATION**

**Observation of the effect of a therapeutic education programme for patients with diabetes in a socio-economically deprived area of the city of Rennes organised by a multi-professional primary care practice.**

**Quantitative and retrospective comparative study on 210 patients.**

| **Research category**: RESEARCH OUTSIDE THE JARDE LAW – BASED ON EXISTING DATA | |
| --- | --- |
| **Supervisor:** Rennes 1 University represented by its current legal representative  **Data Protection Representative:** dpo@univ-rennes1.fr | **Coordinating investigator:**  **Dr ALLORY Emmanuel**  Department of general practice,  University of Rennes 1  emmanuel.allory@chu-rennes.fr  02.23.23.49.68 (Secretariat General practice Department) |

| Person in charge of data treatment and in contact with the participant: AJROUCHE Sarah  Tel: 06 88 34 73 02  Mail: sarah.ajrouche@hotmail.fr |
| --- |

| **This document is addressed to the participant** |
| --- |

| **To be completed by the person qualified to give the information** |
| --- |
| Name / Surname of the participant: …………………………………………………………………  Information letter given: 01/12/2021  **By the investigator physician**:  SURNAME: AJROUCHE Name: Sarah  Address: 21 rue nantaise  35000 Rennes  Mail : sarah.ajrouche@hotmail.fr  Telephone: 06 88 34 73 02 or 02.23.23.49.68 (secretariat of the General practice Department) |

Dear Madame, Sir,

Dr Emmanuel ALLORY who works at the multi-professional primary care practice of Villejean Beauregard and is affiliated to the general practice department at the School of Medicine of Rennes, wishes to inform you about the following PhD project in general medicine: **Observation of the effect of a therapeutic education programme for patients with diabetes in a socio-economically deprived area of the city of Rennes on diabetes control, organised by a multi-professional primary care practice.** With the present information letter, we collect your consent to use and collect data from your health record.

The General Practice Department of the School of Medicine of Rennes – Rennes 1 University supervises the organisation of this study, and is responsible of the treatment.

The purpose of this letter is to inform you of the study objective and its implications and to ensure that you do not object to the collection of these data. However, if there are any points that are unclear after reading this information letter, or if you need additional information, please do not hesitate to contact the study investigator physician, whose contact information is provided above.

If you are under curatorship, guardianship or safeguard of justice, you cannot participate in the study, and we ask you to inform the investigator physician about it without any delay.

1. **OBJECTIVE OF THE STUDY**

Type 2 diabetes is a chronic disease that concerns approximately 3 million people in France, or 4.8% of the population. The prevalence and severity of this disease is more important among populations with a low socio-economic level. Particularly, the prevalence of diabetes is twice as high among CMU beneficiaries.

Therapeutic patient education is an intervention that has proven its value for improving some indicators by helping patients to become more autonomous in order to improve their quality of life. However, these programmes are not well developed in primary care, and patient participation in therapeutic patient education programmes is low.

In Rennes, the Villejean area, a socio-economically deprived area of the city, is particularly marked by social inequalities in health. The health professionals in this area have joined together to form a multi-professional primary care practice.

In March 2017, as part of the health project of this multi-professional primary care practice, health professionals trained in therapeutic patient education developed a multi-professional programme for patients with diabetes. This programme was launched in March 2017, and its content was approved by the Agence Régionale de Santé (Regional Health Agency). Its interest due to its proximity with the patients’ living and with the healthcare actors working in this area and also due its potential for increasing social contacts has been shown by qualitative studies that collected and analysed data on the population’s expectations. However, we have not found any international study on the effect of a therapeutic education programme carried out by a primary care team on the patients' diabetes control. Moreover, the available literature data are contradictory.

We want to carry out a quantitative study to assess the link between participation in this therapeutic education programme and improvement in diabetes control.

The study will be carried out using data collected from your health record at your primary care practice (computerised or paper-format), without directly implicating you and without any effect on your future care.

The study duration is from November 2015 to November 2019. We hope to be able to analyse data from approximately two hundred and ten patients.

Inclusion criteria:

- Age > 18 years

- Type 1 or type 2 diabetes

- Consent to participate in the study

Non-inclusion criteria:

- Gestational diabetes

- Adult under legal protection (safeguard of justice, curatorship, guardianship), persons deprived of freedom.

1. **STUDY IMPLEMENTATION**

As the analysis is retrospective, there will be no impact of the study on your care.

What data are collected and coded?

- Year of birth, sex, socio-professional category

- Medical history

- Biometric parameters (weight, height, blood pressure)

- Laboratory test results

- Usual treatments

These data will be taken from your health record. Only data already collected will be recorded. We will ensure that all data are anonymised, which means that it will not be possible to link your identity to the data we collect for the study.

At the end of the study, and at your request, you can be informed of the study overall results by the investigator physician.

1. **VOLUNTARY PARTICIPATION**

You are free to refuse to participate in this study at any time, without incurring any prejudice as a result. In this case, you must inform the investigator physician of your decision.

If you do not object within 3 weeks, your data will be collected and analysed.

1. **CONFIDENTIALITY AND PERSONAL DATA UTILISATION**

Within the framework of the research in which we propose you to participate, and the objective of which addresses criteria of public interest, your personal data will be transmitted, treated and analysed with regard to the objectives that were presented to you.

All this information will be processed in a coded form so as not to reveal your identity, to guarantee their confidentiality and your anonymity, without mentioning your name and surname.

The doctor involved in this research is subject to professional secrecy, as it is your treating physician.

The data collected, strictly necessary for the research, will be transmitted to the research sponsor or its partners (persons or companies acting on its behalf), in France.

These data may also be transmitted to the French authorities.

The collected data will be kept by the person in charge up to 2 years after the last publication of the results of the study, or in case of absence of publication, until the signature of the final study report. Then, they will be stored on paper or computer for a maximum period of 20 years after the study end.

The DMG of Rennes is the data controller within the meaning of the European Regulation 2016/679, because it determines the purposes and means of data processing in the context of this study. Article 9 of this regulation allows processing special categories of data, including health data.

**Sharing anonymised personal data**

In compliance with the current regulations and in the spirit of sharing the common good that research data represent, the availability of your anonymised personal data is envisaged.

If you wish to participate in the study, but do not wish to share your de-identified personal data, please inform the sponsor in writing.

1. **PERSONAL RIGHTS**

In accordance with the provisions of the French data protection and privacy law (law of 6 January, 1978 as amended) and the European Regulation 2016/679 of 27 April, 2016, you have the right to access, portability, rectification, deletion and limitation of your personal data.

You also have the right to object to the transmission of data covered by professional secrecy that may be used in this study and processed. Exercising this right will result in the termination of your participation in the trial. If your objection to the transmission of data concerns only the sharing of anonymous personal data, your participation in the study will be maintained.

You may also access all your medical information directly or through a qualified person of your choice, in accordance with the provisions of article L 1111-7 of the French Public Health Code.

These rights can be exercised with the investigating physician who is following you in the framework of the study and who knows your identity.

For any complaint relating to the processing of your health data, you can contact the Commission Nationale Informatique et Liberté (CNIL; National Commission for Data Protection and Liberties) (<https://www.cnil.fr/fr/webform/adresser-une-plainte>).

1. **LEGAL ASPECTS**

**The Ethics Committee of the Rennes CHU (CER)** evaluated this project and gave its accord to its implementation.

This study is within the framework of the “Reference Methodology MR-004” established by the **Commission Nationale Informatique et Liberté (CNIL; National Commission for Data Protection and Liberties)**. The department of general medicine of Rennes 1 University, as the study sponsor, signed a compliance commitment to this “Reference Methodology” ensuring that the treatment of personal data follows the CNIL requirements.

**If you decide to participate in this study, please keep this information letter**

**To be completed and sent back by the participant in case of refusal to participate**

**Copy the following sentence and add your surname and name:**

 I, undersigned, Name/Surname refuse to participate in this study “**Observation of the effect of a therapeutic education programme for patients with diabetes in a socio-economically deprived area of the city of Rennes on diabetes control, organised by a multi-professional primary care practice”.**

…………………………………………………………………………………………………………………………..

…………………………………………………………………………………………………………………………..

………………………………………………………………………………………………………………………….

Signature/Date: ……………………………………………………………………………………………………..

# Supplementary file 5 - Agreement of the University Hospital Ethics Committee

| 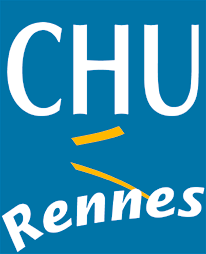 |
| --- |
| **comite d’ethique du CHU** |

Dr Vincent MOREL

Praticien Hospitalier

🕾 02 99 87 35 53

Fax 02 99 87 35 54

[comite.ethique@chu-rennes.fr](mailto:comite.ethique@chu-rennes.fr)

Doctor Sarah AJROUCHE

Faculté de médecine de Rennes 1

2 Avenue du Pr Léon Bernard

35000 RENNES

Opinion n° 21.77-2

Rennes, 14/06/21

Dear Colleague,

After having examined the research project

**“Observation of the effect of a therapeutic education programme for patients with diabetes in a socio-economically deprived area of the city of Rennes on diabetes control, organised by a multi-professional primary care practice.**

**Quantitative retrospective study on 75 patients.”**

The Rennes CHU thinks that this research project does not violate medical ethics.

This opinion of the Ethics committee has been unanimously issued by the present members.

Wishing you a good reception.

Sincerely

For the ethics committee

Vincent MOREL

# Supplementary file 6 – Post-intervention Data

|  | **Intervention group (n=69)** | **Non-intervention group (n=138)** | ***p-value*** |
| --- | --- | --- | --- |
| **Clinical data** | | | |
| Weight in Kg (missing EG n=1, NEG n=6)  BMC in kg/m² (missing EG n=1, NEG n=19)  SBP in mmHg (missing EG n=2, NEG n=3)  DBP in mmHg (missing EG n=2, NEG n=3) | 83.1 ±15.8  31.4 ±6,4  138.2 ±15.3  79.9 ±13.6 | 84.2 ±17.3  31.3 ±5.6  134.7 ±13.1  77.8 ±9.5 | *p=0.64*  *p=0.92*  *p=0.09*  *p=0.19* |
| **Long-term pharmacological treatment** (missing n=1) | | | |
| Metformin  Other oral treatment  GLP-1 analogue  Insulin  ACEI or ARA II  Statins | 56 (82.4%)  29 (42.6%)  15 (22.1%)  28 (41.2%)  42 (61.8%)  31 (45.6%) | 111 (81.0%)  45 (32.8%)  15 (10.9%)  29 (21.2%)  75 (54.7%)  64 (46.7%) | *p=0.82*  *p=0.17*  *p=0.03*  *p=0.01*  *p=0.34*  *p=0.88* |
| **Laboratory data** | | | |
| HbA1c in % (missing n=3)  LDLc in mmol/L (missing EG n=15, NEG n=30)  GFR in mL/min/1,73m² (missing n=EG n=8, NEG n=11)  Microalbuminuria test performed (missing n=0)  Positive microalbuminuria (missing n=EG n=14, NEG n=42) | 7.6 ±0.9  2.5 ±1.1  82.9 ±20.5  55 (79.7%)  16 (29.1%) | 7.5 ±1.2  2.7 ±1.1  84.6 ±21.9  96 (69.6%)  22 (22.9%) | *p=0.83*  *p=0.23*  *p=0.61*  *p=0.12*  *p=0.40* |
| **Specialised follow-up** | | | |
| Ophthalmological consultation (missing n=EG n=19, NEG n=25) | 36 (72.0%) | 43 (38.1%) | *p<0.01* |

Continuous variables are expressed as mean (± standard deviation) and were compared with the Student’s *t* or Mann-Whitney Wilcoxon test. Categorical variables are expressed as numbers (percentage) and were compared with the Chi2 or Fisher’s exact test.

EG: exposed group, GLP-1: glucagon-like peptide 1. ACEI: angiotensin converting enzyme inhibitor. ARAII: angiotensin II receptor antagonist. BMI: body mass index. SBP: systolic blood pressure. DBP: diastolic blood pressure. HbA1c: glycated haemoglobin fraction A1c. LDLc : low density lipoprotein c. GFR: glomerular filtration rate, NEG: non exposed group. Positive microalbuminuria: defined as albuminuria ≥ 30mg/24h or albuminuria/creatinuria ratio ≥ 30mg/g.

A p value <0.05 was considered significant.
